# Hepatotoxic combination effects of three azole fungicides in a broad dose range

**DOI:** 10.1007/s00204-017-2087-6

**Published:** 2017-10-16

**Authors:** T. Heise, F. Schmidt, C. Knebel, S. Rieke, W. Haider, I. Geburek, L. Niemann, P. Marx-Stoelting

**Affiliations:** 1 German Federal Institute for Risk Assessment (BfR), Berlin, Germany; 2 Institute for Animal Pathology, Berlin, Germany

**Keywords:** Mixture toxicity, Triazoles, Liver, AOP

## Abstract

Single active substances of pesticides are thoroughly examined for their toxicity before approval. In this context, the liver is frequently found to be the main target organ. Since consumers are generally exposed to multiple residues of different active substances via the diet, it is important to analyse combinations of active substances for potential mixture effects. For the (tri-)azoles, a group of agricultural fungicides and antifungal drugs, combination effects on the liver are likely because of a similar mode of action. Hepatotoxic effects of mixtures of two triazoles (cyproconazole and epoxiconazole) and an imidazole (prochloraz) were investigated in a 28-day feeding study in rats at three dose levels ranging from a typical toxicological reference value to a clear effect dose. Test parameters included organ weights, clinical chemistry, histopathology and morphometry. In addition, molecular parameters were investigated by means of pathway-focused gene expression arrays, quantitative real-time PCR and enzyme activity assays. Effects were compared to those caused by the individual substances as observed at the same dose levels in a previous study. Mixture effects were substantiated by increases in relative and absolute liver weights, histopathological findings and alterations in clinical chemistry parameters at the top dose level. On the molecular level also at lower dose levels, additive effects could be observed for the induction of several cytochrome P 450 enzymes (*Cyp1a1, Cyp2b1, Cyp3a2*), transporters (*Abcb1a, Abcc3*) and of genes encoding for enzymes involved in fatty acid or phospholipid metabolism (*Ppargc1a, Sc4* *mol*). In most cases, treatment with mixtures caused a more pronounced effect as compared to the individual substances. However, the assumption of dose additivity was in general sufficiently conservative to cover mixture effects observed under the conditions of the present study.

**Electronic supplementary material:**

The online version of this article (doi:10.1007/s00204-017-2087-6) contains supplementary material, which is available to authorized users.

## Introduction

Humans can be exposed to an enormous number of possible mixtures of chemicals via different routes in their daily life (Kienzler et al. 2016). In the past years, evidence has accumulated that exposure to multiple chemicals may lead to combination effects causing a higher toxicity as expected from knowledge about the single substances and thereby potentially affecting human health (Cedergreen 2014; Kortenkamp et al. 2009). Pesticides followed by pharmaceuticals and personal care products are reported to dominate the observed mixture effects in the environment (Tang et al. 2014), and the occurrence of potential combination effects of pesticides is an area of increasing concern for the public and regulatory authorities. Since substances are tested for regulatory purposes on an individual basis at generally high dose levels, there are only limited data available on potential mixture effects especially in the consumer-relevant low dose range. Hence, experimental analysis of mixture effects especially by comprehensive in vivo methods is regarded a key challenge by several EU reports (Kortenkamp et al. 2009).

One focus of research on mixture effects of pesticides has been endocrine disruption (Hass et al. 2012; Orton et al. 2011; Seeger et al. 2016). Since the liver is the main target organ of many pesticides, potential hepatotoxic mixture effects are of high relevance. Surprisingly, only few in vivo experiments have been published which address the issue, and even fewer focus on pesticides (Ito et al. 1995, 1996; Rignall et al. 2013; Wang et al. 2014; Yoshida et al. 2011).

One group of pesticides with high production volumes frequently occurring as residues in and on foods is the triazole group of fungicides. This group has been proposed by EFSA to form a cumulative assessment group (CAG) for chronic exposure based on hepatotoxic abilities (EFSA 2009). Triazole fungicides inhibit a fungal cytochrome P 450 (CYP) enzyme, and therefore, it is plausible that a considerable part of their toxic effects in mammals is due to an unspecific inhibition of mammalian CYP enzymes. As some CYPs are essential for the biosynthesis of cholesterol or steroid hormones, there are several reports, which show that triazoles such as myclobutanil or triadimefon interfere with CYPs important for biosynthesis of sex steroids like CYP17A1 or CYP19A1 (aromatase) leading to disturbances in the biosynthesis of estradiol or testosterone (Goetz and Dix 2009a; Trosken et al. 2004; Zarn et al. 2003). Moreover, effects of triazoles, which are used as antifungal drugs in humans (such as ketoconazole), on degradation of all-trans retinoic acid involving CYP26, have been reported (Menegola et al. 2006; Vanden Bossche et al. 1988). While they inhibit certain CYP enzymes in endocrine target tissues on the one hand, triazoles are also known inducers of hepatic CYPs by activation of nuclear receptors.

For their approval as active substances for pesticidal use as well as on a molecular level, the hepatotoxic properties of several individual triazoles have been analysed in a number of standard toxicity tests (Dewhurst and Dellarco 2004; EFSA 2008a, b, 2009; Goetz and Dix 2009a, b; Tully et al. 2006; Wolf et al. 2006). These studies elucidated that treatment of rodents with high doses of triazoles causes an increase in liver weight and in long-term toxicity studies some triazoles like cyproconazole or epoxiconazole also cause hepatocellular tumours (EFSA 2008a, 2010; Hester et al. 2012). It has previously been demonstrated that triazoles regulate several target genes of the constitutive androstane receptor (CAR) but do most likely also activate other nuclear receptors like the pregnane-X-receptor (PXR) (Goetz et al. 2006; Heise et al. 2015; Hester et al. 2006, 2012; Marx-Stoelting et al. 2017; Nesnow et al. 2009; Peffer et al. 2007). For cyproconazole, direct involvement of CAR in hepatotoxicity was shown by use of respective CAR knockout model and respective humanised mice (Marx-Stoelting et al. 2017; Peffer et al. 2007). Additionally global gene expression analyses show similarities in the expression profiles of some triazoles like propiconazole and the well-known CAR activator phenobarbital (Currie et al. 2014). The imidazole fungicide prochloraz has also been demonstrated to induce hepatotoxicity in rats and mice, e.g. an increase in liver weight and tumours at high dose levels in long-term regulatory studies (EFSA 2011). However, despite structural similarities with other azole fungicides the activation of the aryl-hydrocarbon receptor (AhR) by prochloraz has been assumed as an alternative mechanism to CAR or PXR activation (Long et al. 2003) and this hypothesis has also been investigated in rodent liver and other tissues (Heise et al. 2015; Rieke et al. 2014).

The protocol for the present study was based on a regulatory guideline (OECD TG407), but additional molecular methods were performed, including toxicity pathway-focused low-density gene expression arrays with subsets of marker genes previously identified as triazole related (Goetz and Dix 2009a; Tully et al. 2006; Ward et al. 2006).

Despite the knowledge on hepatotoxic effects caused by individual triazole- or imidazole-fungicides and the proposal to group these fungicides into a CAG (EFSA 2009) combination effects on liver have not been analysed in vivo so far. Here we report the results of a 28-day feeding study, where cyproconazole (C), epoxiconazole (E) and prochloraz (P) were applied as one binary (CE) and one ternary mixture (CEP) to male rats at three dose levels and compare the results with effects seen in a 28-day feeding study where cyproconazole (C), epoxiconazole (E) and prochloraz (P) were fed as individual substances (Heise et al. 2015). Dose selection was based on the no-observed-adverse-effect levels (NOAELs) obtained in regulatory studies used in the frame of the approval procedure of the individual substance and ranged from a dose equivalent to a typical toxicological health-based threshold level, i.e. maximum allowed realistic exposure (NOAEL/100) up to a dose level supposed to show toxic effects (10xNOAEL). Endpoints analysed included organ weights, clinical chemistry and histopathology and in addition molecular parameters as gene expression analysis and enzyme activity assays.

In this paper, we report the findings obtained with the binary and ternary mixture and compare them to the effects observed after administration of the individual substances alone.

## Materials and methods

### Animal experiments

The animal experiments were performed as previously described (Heise et al. 2015; Schmidt et al. 2016). Briefly, healthy 6–7-week-old male Wistar rats (Crl:Wi) from a SPF colony (Charles River Laboratories, Sulzfeld, Germany) were acclimatised to conditions of the animal facility for 2–3 weeks and continuously monitored for mortality, clinical signs or behavioural alterations. At the beginning of the feeding study, all animals were 9 weeks old. Ten animals each were randomly allocated to treatment groups for the single substances or the combinations, respectively, and group caged in Makrolon cages (type IV with heightened cover). Animals were weighed before the beginning of the experiment, once per week during the experiment and before the animals were killed. Blood was sampled from the animals 3 days before the start of the experiment and before the animals were killed. All animals had ad libitum access to azole-supplemented or untreated phytoestrogen-free diet (R/M-H V155, Ssniff, Soest, Germany) and filtered tab water. Food consumption was measured daily by weighing the feed for each cage group individually. Animals were checked daily for clinical signs and mortality. The experiment and parameters analysed were in general accordance with OECD TG407, except that organs isolated and analysed were limited and the experiment was conducted with males only, since males were shown to be slightly more sensitive in previous studies used within the approval procedure for the respective fungicides. In this publication, the scope is limited to mixture effects in the liver. Further experimental details and results are given elsewhere (Heise et al. 2015; Rieke et al. 2017; Schmidt et al. 2016).

### Test substances

Test substances of technical grade were obtained directly from the producing companies. Cyproconazole (CAS no. 94361-06-9, Batch no. CHF1E00042, purity 96.8%) was purchased from Syngenta (Basel, Switzerland), epoxiconazole (CAS no. 133855-98-8, Batch no. 8563, purity 97.0%) and prochloraz (CAS no. 67747-09-5, Batch no. COD-000718, purity 98.0%) were supplied by BASF (Ludwigshafen, Germany). The substances were mixed into the rodent diet at different concentration levels by a solvent-free procedure by Ssniff (Soest, Germany). Dose selection was based on the no-observed-adverse-effect levels (NOAELs) obtained in regulatory studies used in the frame of the approval procedure of the individual substance: NOAEL/100: 0.9 and 1 ppm, NOAEL: 90 and 100 ppm, NOAELx3: 270 and 300 ppm, NOAELx10: 900 and 1000 ppm. For the experiments using mixtures of substances, dose levels were added up; thus, a range from 1.9 up to 1900 ppm for the binary mix I (cyproconazole and epoxiconazole) and 2.9 up to 2900 ppm for the ternary mix II (cyproconazole, epoxiconazole and prochloraz) was applied (see Supplementary Table 1 for details). Concentration and stability of test substances in the rodent diet was checked by SGS Fresenius (Berlin, Germany) by multi-method according to ASU L. 00.00-115 LC under GLP conditions. Accordingly, control diet was checked for the absence of pesticides, especially (tri-)azole fungicides, to ensure the quality of the negative control. For details on target concentration and achieved concentrations of test substances (see Schmidt et al. 2016).

### Sacrifice and gross pathology

After 28 days of treatment, rats were deeply anaesthetised with Sevofluran (Abbot, Germany), cardial blood was sampled by use of a 21G Sangocan blood sampling canule (Kabe, Germany) and animals were finally killed in 95% CO_2_ and 5% O_2_. Necropsy was performed by an accredited animal pathologist, and livers were isolated, evaluated and weighed. Livers were then divided into several parts: one small part of the lobus lateralis sinister was kept for molecular analysis and frozen in liquid nitrogen, while another (larger) part became subject to histopathological examination.

### Histopathology and morphometry

Directly after isolation, livers were partially frozen on dry ice for subsequent molecular analysis and partially (lobus lateralis sinister) fixed in a 5% formaldehyde solution (Sigma-Aldrich, Germany) for subsequent paraffine embedding (Leica, Germany), microtomy and staining with haematoxylin and eosin (H&E) (Bio-Optica, Italy). Materials obtained from H&E-stained liver slices (2 µM thick) were evaluated microscopically at magnifications from 50× to 400× according to standard principles of the Society for Toxicopathology (STP 2010). Two slices per animal were evaluated independently.

In addition, two slices per animal were stained for the detection of changes in proliferation and apoptosis. For detection of proliferative changes, liver slices were stained with Ki67 antibody, dilution 1:800 (clone EP 5, Epitomics, USA) and for detection of apoptosis, cleaved caspase 3 staining was performed by use of a polyclonal antibody, dilution 1:200 (Zytomed Systems GmbH, Germany).

Morphometric examination was based on cell size measurement of hepatocytes surrounding the central veins, since the observed hypertrophy was limited to the centrolobular area of the liver. Microscopic observation was conducted using a microscope Axio observer from Zeiss (Jena, Germany) at a magnification of 100× to 200×. Liver slides of cyproconazole, epoxiconazole and prochloraz (NOAEL, NOAELx3, NOAELx10) and the corresponding controls were inspected. In addition, both mixtures were examined at the dose levels NOAEL and NOAELx10. Five animals per treatment group were used. Pictures of eight central veins were taken, and the cell size (area) of at least ten cells surrounding the central veins was measured using the AxioVision software (version 4.7).

### Clinical chemistry

The following parameters were analysed: glucose, bilirubin, alkaline phosphatase, aspartate amino transferase, arginine amino transferase, γ-glutamyl transferase (γ-GT, bile acids, cholesterol, creatinine, urea, total protein, albumin, sodium, potassium, Quick, prothrombin time (PTT)). Clinical chemistry was analysed by the Institute for Veterinary Diagnostics (Berlin, Germany) according to institutional standard operation procedures under GLP conditions on a Cobas 6000 c501 analyser (Roche, Switzerland).

### Molecular analysis

RNA was isolated from liver tissues frozen in liquid nitrogen using TRIzol reagent according to the manufacturers’ protocol (Invitrogen, Carlsbad, CA, USA). RNA was further purified using a RNA purification kit (Qiagen, Hilden, Germany). For gene expression analysis via quantitative real-time PCR (qRT-PCR) quality and quantity of RNA samples was controlled with a Nanodrop spectrophotometer (peqLab, Erlangen, Germany). Reverse transcription of 2 µg RNA was performed using High-Capacity cDNA Reverse Transcription Kit and protocol (Applied Biosystems, Darmstadt, Germany). qRT-PCR was performed of 40 ng cDNA on an ABI 7900HT instrument (Applied Biosystems, Darmstadt, Germany) using Maxima SYBR Green/ROX qPCR Mastermix (Life Technologies, Carlsbad, USA) and 0.25 µM primers (Eurofins, Luxemburg) (see Supplementary Table 2). Results were checked for significance on SigmaPlot for Windows software (version 11.0, Systat Software Inc. 2008). For low-density array experiments, the quality of the purified RNA was determined with a bioanalyzer (Agilent Bioanalyzer, Santa Clara, USA) following the manufacturers' standard protocol, and samples with an RNA-integrity-number of 8-10 were admitted into the experiments. 2 µg RNA was reversely transcribed to cDNA by RT^2^ First Strand cDNA Kit according to protocol (Qiagen, Hilden, Germany). RT^2^ profiler PCR array drug metabolism (rat, # PARN-002Z, SA Bioscience, Hilden, Germany) and molecular toxicology pathway finder (rat, # PARN-3401ZE, SA Bioscience, Hilden) were used for sample analysis on an ABI 7900HT real-time PCR system (Applied Biosystems, Darmstadt, Germany). Array evaluation was performed using the web-based RT^2^ Profiler Array Analysis System 4.0.

### Enzyme activity assays

Liver microsomes were isolated in a 250 mM sucrose buffer (Merck, Darmstadt, Germany) by differential centrifugation at a final speed of 100,000*g* for 1 h. Microsomal *O*-dealkylations of 7-ethoxyresorufin (EROD), 7-pentoxyresorufin (PROD) and 7-benzoxyresorufin (BROD) were measured to detect enzyme activity of CYP1A1, CYP2B1 and CYP3A1/2 using resorufin as standard (reagents obtained from Sigma-Aldrich, Basel, Switzerland). The assay was performed at 37 °C in a KH_2_PO_4_/K_2_HPO_4_ buffer at pH 7.4 for 2 h. Measurement of resorufin was conducted on a Tecan Plate Reader (Tecan, Infinite M200Pro).

### Statistics

Statistical analysis was performed using parametric and nonparametric standard tests by SigmaPlot for Windows software (Version 11.0, Systat Software Inc. 2008). Data were analysed for normal distribution using Shapiro–Wilks or Kruskal–Wallis tests and for homogeneity of variance with *p* < 0.05. If data were shown to be normally distributed and variances were considered to be equal, parametric tests were used for subsequent statistical analysis (two-sided *t* test to compare two groups). If nonparametric tests had to be applied, rank-sum tests according to Mann–Whitney were used to compare two groups. Statistical significance was only assumed if *p* < 0.05 (*) or *p* < 0.01(**).

## Results

### Clinical observations

There was no mortality, and all rats remained in good health throughout the study. Body weight and in particular weight gain were compromised by administration of all three test substances and even more pronounced by the combinations at the top dose levels, see Schmidt et al. (2016) for details. At the lower dose levels, there were no adverse effects on body weight and its development, on food consumption or utilisation with any of the substances or the combinations.

### Clinical chemistry

Clinical chemistry parameters were analysed prior to the beginning of the 28-day feeding study and at the end of the study. No significant deviations from controls were observed at the beginning of the study confirming the health status of the animals (data not shown). After 28 days of treatment with the individual substances, all parameters analysed were not different from controls with the only exception of a significant increase in cholesterol concentration in the group receiving epoxiconazole at the top dose level. With the combinations, in contrast, additional parameters were affected. In particular with regard to liver, a small but significant increase in γ-GT activity was observed at the top dose level of both combinations, confirming increased liver toxicity by the mixtures. While γ-GT activity was 0 at the lower dose levels of mix I and mix II and at all dose levels for the individual substances at the top dose level of mix II an activity of > 0.03 µkat/L was detected. However, no other parameters that may indicate alterations of liver function (e.g. ALAT, ASAT, albumin, bilirubin) were significantly altered (i.e. increased above controls).

### Pathology and liver weights

**Fig. 1:**
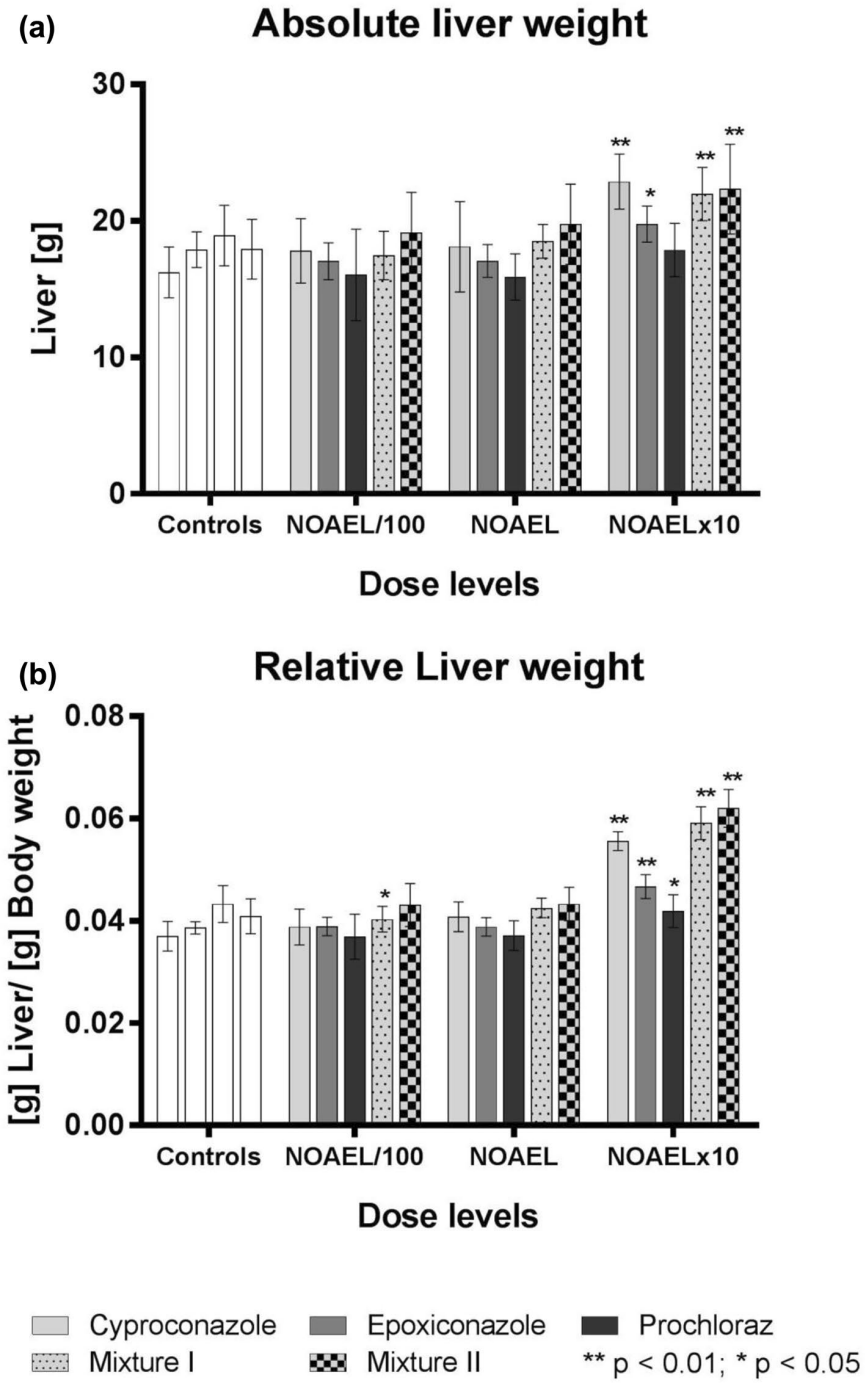
Organ weights: **a** absolute liver weights in g. **b** Relative liver weights in g liver per g body weight. Error bars represent standard deviations for the respective dose level. Controls had an average relative liver weight of 0.04 g liver/g bw. Statistically significant differences compared to controls are indicated with (*) if *p* < 0.05 and with (**) if *p* < 0.01

Treatment-related alterations in absolute and relative liver weights have already been reported for the individual substances (see Heise et al. 2015) for details) and are summarised in Fig. 1. In brief, liver weight was significantly altered in response to treatment with the highest dose levels (NOAELx10) of cyproconazole, epoxiconazole and the substance mixtures I and II. Although cyproconazole and epoxiconazole as single substances individually induced an increase in absolute liver weight (28 and 11%, respectively) the mixture (mix I) containing the same dose of this substances increased the absolute liver weight only up to 16%. The mixture containing prochloraz (mix II) in addition showed an effect (increase of 25%) comparable to that of the most potent substance in the mixture, i.e. cyproconazole alone. The more pronounced effect of the mixtures on relative liver weight (increase up to 47%) was attributed to lower body weight. No gross lesions in the liver were observed with any of the mixtures.

### Histopathology

**Fig. 2:**
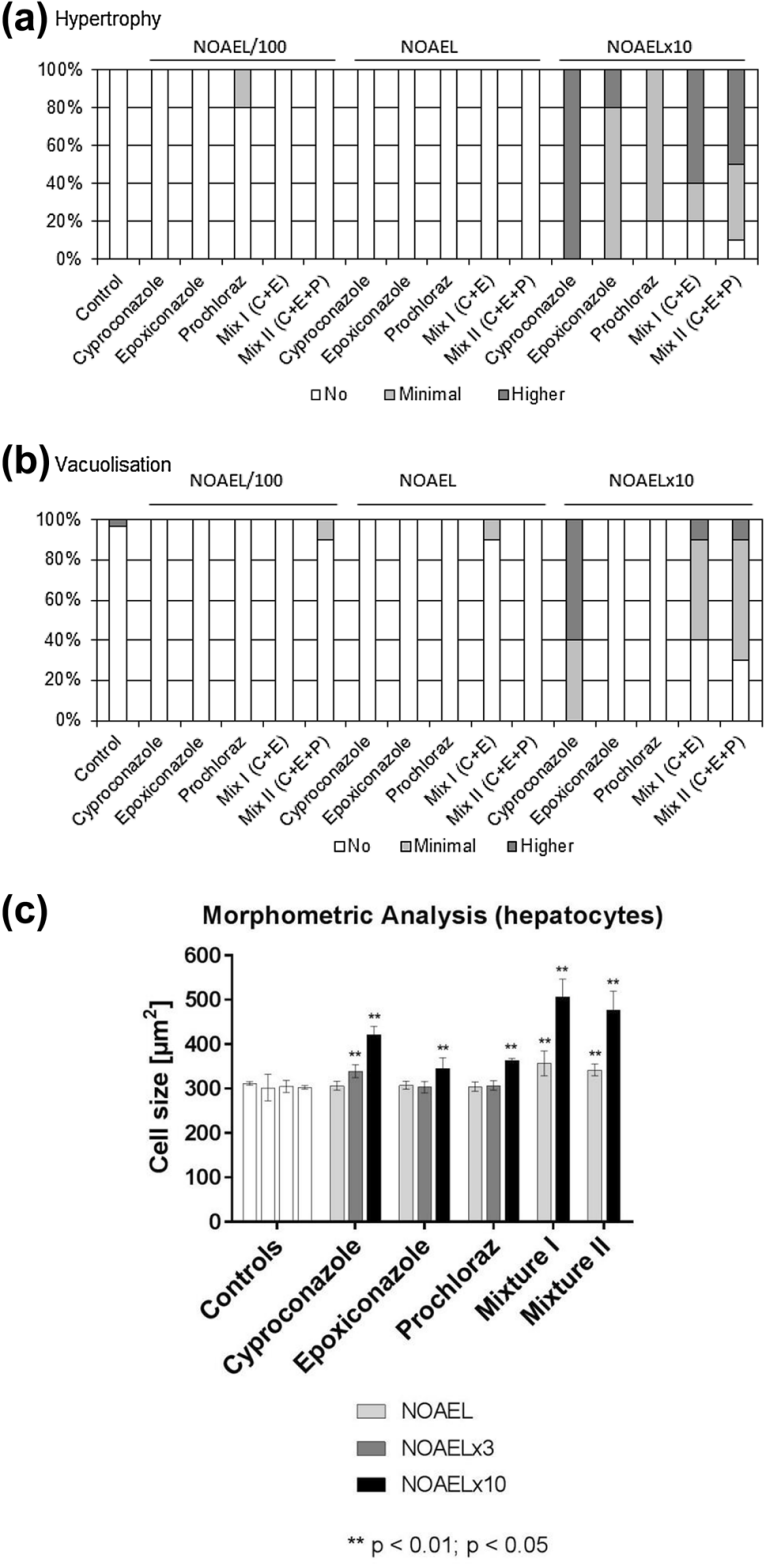
Per cent of livers per substance and dose group showing **a** hypertrophy or **b** vacuolisation. The effect was graded to be either minimal or higher. **c** Morphometric analyses of hepatocytes surrounding the central veins. Statistically significant differences as compared to controls are indicated with (*) if *p* < 0.05 and with (**) if *p* < 0.01. Error bars represent standard deviations for the respective dose levels

Histopathological observations have already been reported for the individual substances (Heise et al. 2015; Schmidt et al. 2016). In brief, treatment-related effects observed in livers included vacuolisation and hypertrophy of hepatocytes and were in general limited to the highest dose level (NOAELx10) with cyproconazole showing the most pronounced effects. These findings were confirmed with the combinations. 80–100% of the animals receiving the maximum dose of single substances or their mixtures showed hypertrophy of hepatocytes, while 100% of the rats treated with cyproconazole showed vacuolisation this finding was only detected in 60 and 70% of the mix I and mix II treated animals, respectively (Fig. 2a, b).

The analysis of Ki67 staining revealed no induction of proliferation (data not shown) and the analysis of cleaved executioner caspase 3 revealed no significant induction of apoptotic events, for the single substances as well as for the substance mixtures (data not shown).

### Morphometric analysis

The morphometric analysis of hepatocytes at the central vein revealed a dose-dependent increase in their size (Fig. 2c). After exposure to cyproconazole at the intermediate high dose level of 300 ppm (NOAELx3), the size of centrolobular hepatocytes was significantly increased by 9% and at the top dose level of 1000 ppm (NOAELx10) up to 40% in comparison with the corresponding control groups. Cell sizes were also significantly enhanced after exposure to prochloraz and epoxiconazole at their respective top dose levels of 1000 or 900 ppm (by 20 or 14%, respectively). In line with these findings, both mixtures already induced a small but significant increase in cell size at the mid dose level (mixture 1: 17%, mixture 2: 13%). At the maximum dose levels, the size of hepatocytes was clearly more increased than following single exposure to cyproconazole or epoxiconazole. Area increments accounted for 66% (mixture 1) or 58% (mixture 2).

### Cyp gene expression and enzyme activity

**Fig. 3:**
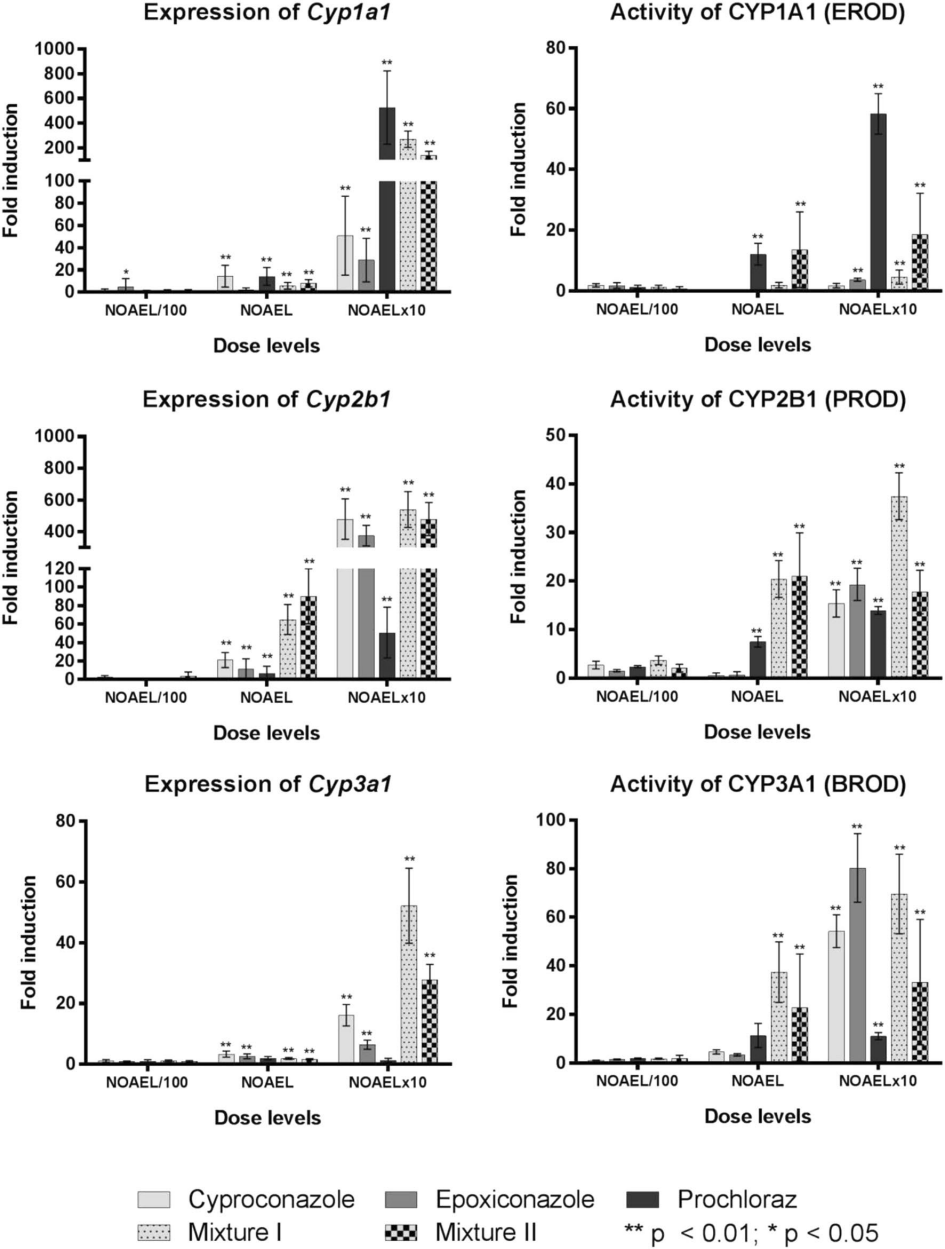
Correlation between gene expression of *Cyp1a1*, *Cyp2b1* and *Cyp3a1* and enzyme activity measured by respective alkoxy-resorufin-*O*-dealkylation at NOAEL/100, NOAEL and NOAELx10. Figures show fold induction relative to the respective controls; error bars represent standard deviations for the respective dose level. Statistically significant differences as compared to controls are indicated with (*) if *p* < 0.05 and with (**) if *p* < 0.01

As for the individual substances also for the mixtures, a panel of genes coding for xenobiotic metabolising enzymes was analysed in the whole dose range by quantitative real-time PCR and respective enzyme activity was analysed by alkoxy-resorufin-*O*-dealkylation assays. Results for *Cyp1a1*/CYP1A1 (a target of AhR signalling), *Cyp2b1*/CYP2B1 (a CAR target) and *Cyp3a1*/CYP3A1 (a PXR target) are presented in Fig. 3. The effects on gene expression in response to the exposure with single substances are described in (Heise et al. 2015). For most genes, a dose-dependent increase in expression could be observed with a threshold at NOAEL/100, accompanied by changes in enzyme activity starting at NOAEL or NOAELx10 for the single substances as well as for the mixtures. For mixture I (C&E) a significant increase in mRNA of *Cyp1a1* and *Cyp3a1* could be observed at the highest dose level that was larger than the effect of both single substances in sum; however, the increase significantly induced by the prochloraz containing mix II was smaller than observed for mix I and in the case of *Cyp1a1* even considerably smaller as for prochloraz alone (Fig. 3). At the dose level NOAEL, a significant increase of mRNA of *Cyp1a1* and *Cyp3a1* induced by the mixtures was smaller than the induction observed in response to the single substance. For *Cyp2b1* mRNA, a significant increase following the exposure with mix I and II was at the mid dose level (NOAEL) stronger in comparison with the sum of the single substances (Fig. 3). At the highest dose level, mix I significantly induced a smaller increase of *Cyp2b1* mRNA than the both single substances in sum and the effect of mix II was, moreover, smaller as of mix I and similar to the effect of cyproconazole alone. In general, the induction of gene expression of the analysed Cyps in response to mix II was similar or even smaller than the effect of mix I at the dose levels NOAEL and NOAELx10.

A dose-dependent increase in the activity of the analysed enzymes was observed for mix I. At the mid dose level, the induction of CYP1A1, CYP2B1 and CYP3A1 enzyme activity by the mixture I was stronger than assumed by the sum of the effects of the single substances; however, at the highest dose level a significant increase in enzyme activity of CYP2B1 could be detected which was comparable to the sum of the effects of the single substances, while the increase in CYP1A1 enzyme activity was below the sum of the single effects and the induction of CYP3A1 enzyme activity was even smaller than the increase induced in response to epoxiconazole.

At the dose level NOAEL, mix II induced a significant increase in CYP1A1 and CYP3A1 that corresponds to the summed up induction of the three single substances; however, the induction of CYP2B1 enzyme activity was higher than assumed by the effects of the single substances. At the dose level NOAELx10 in contrast the strength of induction for all enzymes was smaller than the strongest effect observed by one of the single substance applied alone and therefore well below the sum of the single substances. Similar to the patterns observed for gene expression at the dose levels NOAEL and NOAELx10, the effect of mix II on enzyme activity was similar or in most cases smaller in comparison with mix I regarding CYP2B1 and CYP3A1.

While for mix I on the level of gene expression as well as on the level of protein expression effects stronger than the sum of the individual substances could be observed at doses of NOAEL and NOAELx10, only for CYP2B1 enzyme activity an effect higher than assumed by summing up the effects of individual substances could be observed for the prochloraz containing mix II.

### Gene expression array

A low-density array (Toxicity PathwayFinder Array, SABiosciences) was used to analyse the expression of a panel of different genes which are involved in several pathways associated with toxicity. Only the top dose level was used to compare the effects of single substances and substance mixtures regarding the number of affected genes, the strength of gene expression changes and the affected pathways. The results of the array revealed modulation for the expression of a panel of genes: 32% of all analysed genes were significantly altered at least twofold by at least one substance or substance combination (Supplementary Table 3). While the single substances cyproconazole, epoxiconazole and prochloraz induced changes in the expression of 46, 48 and 39 genes, respectively, the substance mixtures affected the expression of a higher number of genes: mix I altered the expression of 76 and mix II of 63 genes. In 86% of the changes in gene expression which were induced by mix I, the observed fold change was higher than the effects induced by one of the single substances alone. Mix II induced in 75% of the altered genes an effect which was stronger than the effect of one of the single substances. However, the observable effects induced by mix II were in 51% of the genes lower than the effects by mix I. Only for some genes (*Asns, Cdkn1a, Cyp1a1, Cyp3a23/3a1, Cyp7a1, Duox1, Duox2, Fmo5, Ly6d, Por*) the gene expression alterations induced by the mixtures were higher than by the sum of effects of the single substances and could be observed for mix I and mix II; e.g. the expression of *Cdkn1a* was significantly downregulated by mix I (0.07-fold) and by cyproconazole and epoxiconazole 0.95- and 0.72-fold, respectively, the expression of *Cyp1a1* was significantly upregulated by the mixtures (mix I: ~ 399-fold, mix II: ~ 690-fold) and by cyproconazole, epoxiconazole and prochloraz ~ 27-, ~ 25- and ~ 405-fold, respectively, and the expression of *Duox1* was significantly upregulated by mix II (~ 52-fold), while cyproconazole, epoxiconazole and prochloraz altered the expression ~ 5-, ~ 12- and ~ 3-fold, respectively, and mix I ~ 14-fold. Overall, the strongest significant changes in gene expression (> 20-fold) were observed for *Abcc3, Aldh1a1, Cyp1a1, Cyp2b2, Cyp3a23/3a1, Duox1* and were induced by individual substances as well as by substance mixtures (see Table 1 for details).

**Table 1:** Effects on gene expression by cyproconazole, epoxiconazole and prochloraz and the two substance combinations as obtained by molecular toxicity pathway finder RT^2^ profiler pcr array for the dose level NOAELx10

Gene	Cyproconazole (mean 2^−∆∆Ct^)	Epoxiconazole (mean 2^−∆∆Ct^)	Prochloraz (mean 2^−∆∆Ct^)	Mixture I (mean 2^−∆∆Ct^)	Mixture II (mean 2^−∆∆Ct^)
	1000 ppm	900 ppm	1000 ppm	1000:900 ppm	1000:900:1000 ppm
Cytochrome P450s and phase I drug metabolism					
*Cyp1a1*	**26.64**	**25.03**	405.03**	398.85**	690.41**
*Cyp1a2*	0.88	1.39	**3.26**	1.90	2.10*
*Cyp2b2*	87.40**	76.21**	17.03**	140.05**	172.46**
*Cyp2c11*	1.35*	2.32**	1.22	1.73*	1.71
*Cyp2c37*	4.20**	5.83**	2.40**	6.41*	5.20**
*Cyp2d4*	**2.45**	3.52*	**0.21**	1.09	**2.85**
*Cyp2e1*	1.21	1.48	1.85	2.08**	1.56
*Cyp3a2*	4.52*	5.06**	2.84*	5.47**	4.63**
*Cyp3a23/3a1*	13.52**	6.52**	2.57**	26.13**	24.10**
*Fmo2*	0.38*	1.20	1.49	2.25*	1.75*
*Fmo5*	0.80	1.22	1.11	2.46*	**2.51**
Cholestasis					
*Abcb11*	1.79**	1.65	1.78**	2.40**	2.10**
*Abcb1a*	7.79**	5.75*	3.45*	12.60**	9.99**
*Abcc1*	1.66*	1.78	1.28	2.52*	1.83*
*Abcc2*	2.69*	3.77*	2.52**	5.39*	3.96**
*Abcc3*	74.16**	71.82*	14.57*	94.70**	100.47**
*Atp8b1*	3.42**	3.98**	3.52**	4.98**	**3.32**
*Cyp7a1*	1.49	**4.70**	5.44*	8.89**	**2.81**
*Dlat*	1.79*	2.33**	1.43*	1.94*	1.79
*Icam1*	2.05*	1.57	1.14	1.66	1.59
*Nup210*	2.02*	1.98	1.11	**2.64**	**2.03**
*Pdyn*	**2.32**	**2.04**	3.92**	**5.42**	**5.82**
Phospholipidosis					
*Abcb1b*	5.89*	**19.42**	**2.46**	**2.21**	4.11*
*Aldh1a1*	22.68**	13.02**	5.04*	22.99**	26.18**
*Asns*	**2.07**	1.21	0.95	9.09*	9.26**
*Ces2c*	6.62**	5.63**	**2.65**	14.95**	14.40**
*Ephx1*	3.66**	4.17*	**2.09**	4.05**	5.42*
*Fxc1*	1.60*	1.57*	1.73**	**2.16**	2.73**
*Hpn*	1.96	2.24*	1.69	1.88	1.33
*Lss*	1.66	1.78*	1.43	2.70**	1.68*
*Manba*	2.79**	1.62*	1.30*	2.69**	2.53**
*Mlx*	1.30	1.60*	1.69*	1.92**	2.04**
*Mrps18b*	1.64**	1.67**	1.74**	1.72*	2.12**
*Nr0b2*	1.25	**3.80**	**4.83**	5.07*	**3.28**
*Por*	4.01**	4.74**	4.17*	11.62**	8.75**
*Sc4* *mol*	2.00*	1.65	2.63**	3.68**	3.48**
*Slco1a4*	3.96**	2.94**	1.78	4.86**	3.74**
*Stbd1*	2.62**	2.75**	2.61**	3.69*	**2.37**
*Ugt1a1*	4.12**	2.99**	1.70**	8.09**	5.91**
*Ugt2b1*	6.60**	6.06**	4.17**	11.16**	11.93*
Steatosis					
*Acaca*	1.58**	3.44**	1.08	2.77*	1.93**
*Cd36*	2.95*	1.46	1.92*	2.41*	3.08**
*Comt*	1.39	1.94	1.11	2.33**	2.15*
*Fasn*	2.74**	4.99**	1.29	1.85	1.97
*Gpd1*	1.98**	2.36**	1.46*	1.92*	1.72**
*Ly6d*	1.12	1.82*	2.57**	**12.20**	8.56*
*Ppara*	1.66	1.89*	1.69	3.82*	2.80*
*Ppargc1a*	3.68**	2.78**	3.48**	7.14**	5.33**
*Scd1*	0.58	1.90	0.18*	0.64	**0.45**
*Tff3*	2.32*	1.60	2.94**	**3.40**	1.86
Others					
*Ahr*	3.07**	3.60**	**2.44**	3.52*	2.97*
*Atm*	2.33*	2.53**	2.66**	2.90**	2.78*
*Cdkn1a*	0.95	0.72	0.23**	0.07**	0.11**
*Duox1*	**5.35**	**11.79**	**2.74**	**13.64**	51.68*
*Duox2*	**2.52**	**5.09**	2.07*	6.31*	12.58*
*Gsta5*	6.60**	**4.59**	3.36*	8.74**	12.61**
*Hspb8*	2.42*	2.88**	2.43**	3.09**	3.06**
*Mdm2*	1.74**	2.01**	1.37*	2.69**	2.71**
*Nploc4*	2.10**	2.58**	2.00**	2.65**	2.53**

Genes were assorted to pathways as suggested by the manufacturer based on a pathway-focused analysis of the genes. Of the 384 genes analysed on the array only those are listed here for which fold induction or repression by more than factor 2 with a significance of *p* < 0.05 was found in any of the treatment groups, and these expression changes are printed bold. In addition, only genes belonging to the pathways cytochrome P450s and phase I drug metabolism, cholestasis, phospholipidosis and steatosis and as well as selected genes from other pathways are presented

**Fig. 4:**
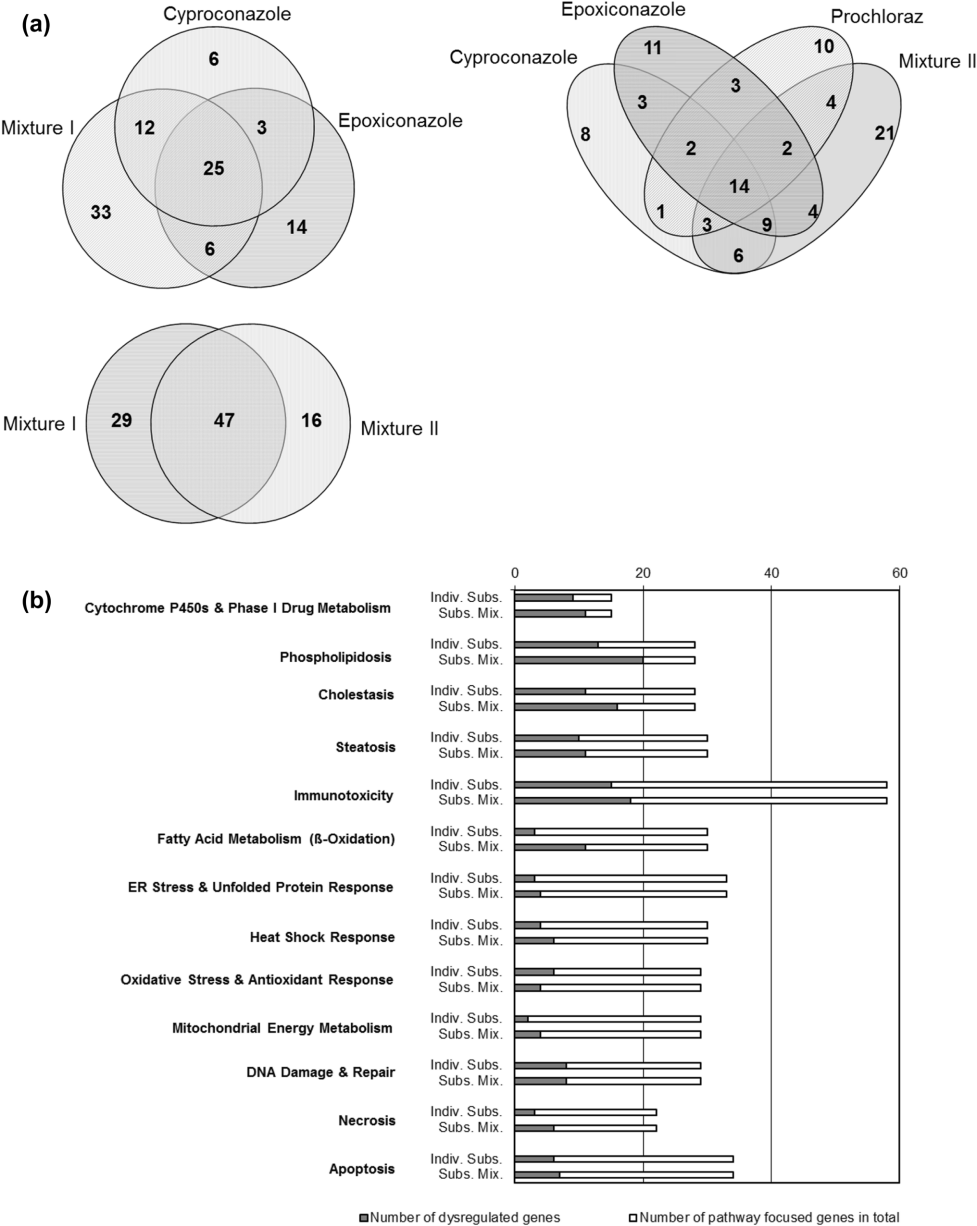
**a** Venn diagrams showing the correlation between individual substances cyproconazole, epoxiconazole and prochloraz and substance mixtures mix I and mix II in the molecular toxicity pathway finder RT^2^ profiler PCR array. **b** Genes analysed using the molecular toxicity pathway finder RT^2^ profiler PCR array were assorted to pathways as suggested by the manufacturer based on a pathway-focused analysis of the genes. Presented are percentages of pathway-focused genes altered by either treatment with individual substances (cyproconazole, epoxiconazole or prochloraz) or substance mixtures (mix I and mix II). For **a** and **b** only genes where the expression was significantly altered greater than twofold with *p* < 0.05 (*t* test) were taken into account for the evaluation

A comparison of the genes altered in their expression by the individual substances and/or mixtures showed that the expression of 25 genes was commonly modified by cyproconazole, epoxiconazole as well as by mix I. Fourteen of these genes were also altered in their expression by prochloraz and the prochloraz containing mix II (*Abcb1a, Abcc2, Aldh1a1, Atm, Cyp2b2, Cyp2c37, Cyp3a2, Cyp3a23/3a1, Hspb8, Nploc4, Por, Ppargc1a, Ugt2b*). Mix I and mix II altered the expression of 47 common genes which corresponds to 62 and 75% of the total number of genes affected by mix I and mix II, respectively (see Venn diagrams in Fig. 4a).

The targeted genes of the molecular toxicology pathway finder array can be assigned to functional groups. A comparison of the effects of the single substances versus the mixtures revealed no difference in the top-scored pathways and confirmed that the analysed (tri-)azoles mainly affect genes which are involved in cytochrome P450s and phase I drug metabolism, phospholipidosis, cholestasis and steatosis (see Fig. 4b).

## Discussion

The aim of this study was to investigate hepatotoxic combination effects of two triazoles (epoxiconazole and cyproconazole) and one imidazole fungicide (prochloraz) in a broad dose range and to compare the outcome of the results obtained for the individual substances to the results obtained for the combinations. Besides a classical toxicological analysis, additional molecular parameters were investigated to check the suitability of these methods for the detection of mixture effects. Another reason was to find out whether and to which extent molecular changes could be associated with adverse outcomes.

Our results suggest that with respect to the adverse outcomes observed on the tissue or organ level, mixture effects were less pronounced than assumed if substances would cause additive effects. Even if liver weight and histopathology were affected slightly more by the combinations than by the individual substances, the increase was less than expected. A reason for this deviation may be due to toxicokinetics: recently, it was shown that simultaneous administration of cyproconazole and epoxiconazole led to a decrease in organ levels of the more potent hepatotoxic compound cyproconazole (Schmidt et al. 2016). This finding was confirmed when cyproconazole, epoxiconazole and prochloraz were administered simultaneously (Schmidt et al. 2016). Consequently, the overall intra-organ concentration of triazoles administered simultaneously was lower than the sum of the intra-organ concentrations of the individual substances when administered alone for mixture I and the more potent substance was showing a reduced level in both mixtures.

Another reason for the limited magnitude of combination effects observed by classical toxicology may be of methodological nature: histopathology does not produce continuous but dichotomous data (hypertrophy is present or not present or, if at all, graded into four categories). Thus, this method is of limited value to quantify combination effects. However, morphometry or clinical chemistry could generally help to overcome this deficiency due to the continuous nature of data obtained by these methods. In our study, this was possible for morphometry only, because only for this method quantifiable results were obtained.

Another methodological problem when analysing combination effects is the limited number of dose levels and especially dose levels causing effects: to model effects and compare them with model curves for dose addition or effect addition (Hadrup et al. 2013) it is generally necessary to analyse several (at least five) effect doses for the individual substances. This is not possible with the limited amount of effect doses in most in vivo experiments including the present study. Here in vitro experiments may help to understand the nature of mixture effects (i.e. if there is dose additivity, effect additivity or interaction). Consequently, a combination of in vitro and in vivo experiments may be necessary to address both: the nature of the combination effect on a selected endpoint like receptor activation and its relevance in vivo.

Our pathway-focused gene expression analysis revealed four toxicity pathways being affected: cytochrome P450s and phase I drug metabolism, steatosis, cholestasis and phospholipidosis. Most interestingly, these pathways were affected by the individual substances in a first experiment (Heise et al. 2015) as well as by the combinations in the present experiment, indicating a high level of reproducibility of molecular effects at least on the pathway level. To focus on the pathway level therefore seems a promising way forward when considering the low level of reproducibility sometimes associated with gene expression analysis (Tan et al. 2003).

Our results indicate that the mode of action remains unchanged, when several compounds of the same group are administered, indicating common molecular targets. While the gene expression patterns and adverse effects associated with the induction of xenobiotic metabolism and steatosis are discussed in detail for the individual substances elsewhere (Heise et al. 2015) gene expression changes associated with cholestasis will be discussed here.

A very important concept to link molecular effects with adverse outcomes is the adverse outcome pathway (AOP) (Villeneuve et al. 2014). In brief, the AOP requires the identification of a molecular initiating event (MIE) causing changes including some key events (KE) on the molecular, cellular and tissue level, ultimately leading to an adverse outcome like steatosis or cholestasis (OECD 2013; Vinken 2013). Essentially, both sides have to be considered: the molecular signature and the adverse effects (Marx-Stoelting et al. 2015). For cholestasis, the MIE is the inhibition of the bile salt export pump and consequently the accumulation of bile salts in hepatocytes (Vinken et al. 2013).This is considered to lead to the activation of several nuclear receptors primarily FXR but also of CAR and PXR. As a consequence molecular changes occur, including up-regulation of *Cyp3a4*, *Mrp3* or *Sult2a1* or downregulation of *Cyp7a1* (Vinken et al. 2013). On the tissue and organism level, an increase in bilirubin and liver enzymes in serum can be observed.

Our results confirm that it is important to consider both: molecular effects and the adverse outcome at the same time, before concluding on a specific AOP: Even if some molecular changes observed in our study point to cholestasis (i.e. alterations in the expression of *Cyp3a1/2* and *Cyp7a1*), the related adverse outcome was not observed. Reason for this may be that the substances themselves induce PXR and CAR and mimic to some extend the molecular signature of cholestasis, while not inducing the adverse effect itself.

A key issue is the question, if consumers are safe, if exposed to combinations of pesticide active substances. Under the conditions of the present experiment, no adverse effects were detected when substances were administered in combination below the NOAEL of the individual substances and only slightly elevated effects were observed at higher dose levels. On the one hand, this may suggest that safety factors and the concept of dose addition were sufficiently protective in the current case. On the other hand, deviations from the assumed dose addition model due to kinetic influences that may well increase the strength of an effect rather than decreasing it as observed in the study reported, support the need to experimentally analyse mixture effects prior to decision making.

## Electronic supplementary material

Below is the link to the electronic supplementary material.

Supplementary material 1 (DOCX 23 kb) Supplementary Table 1: Dose groups, nominal concentrations and relation to the no-observed-adverse-effect level (NOAEL) are presented

Supplementary material 2 (DOC 33 kb) Supplementary Table 2: Primers used to specifically amplify particular rat genes

Supplementary material 3 (DOCX 45 kb) Supplementary Table 3: Effects on gene expression by cyproconazole, epoxiconazole and prochloraz and the two substance combinations as obtained by molecular toxicity pathway finder RT^2^ profiler PCR array for the dose level NOALx10. Of the 384 genes analysed on the array only those are listed here for which fold induction or repression by more than factor 2 with a significance of *p* < 0.05 was found in any of the treatment groups, and these expression changes are printed bold
