# Supplementary material for: Hepatotoxic combination effects of three azole fungicides in a broad dose range
Source: Arch Toxicol. 2017 Oct 16;92(2):859–72. doi: 10.1007/s00204-017-2087-6 (PMC5818588; doi:10.1007/s00204-017-2087-6)
Supplement: Supplementary file 1 — Supplementary material 1 (DOCX 23 kb) Supplementary Table 1: Dose groups, nominal concentrations and relation to the no-observed-adverse-effect level (NOAEL) are presented [file 204_2017_2087_MOESM1_ESM.docx]

**Table 1** Substances, composition of the mixtures, nominal concentrations and relation to the no observed adverse effect level (NOAEL).

| **Test substances** | **Nominal dose level [ppm]** | **Relation to NOAEL** |
| --- | --- | --- |
| **Cyproconazole** | 1 | NOAEL/100 |
|  | 100 | NOAEL |
|  | 1000 | NOAELx10 |
| **Epoxiconazole** | 0.9 | NOAEL/100 |
|  | 90 | NOAEL |
|  | 900 | NOAELx10 |
| **Prochloraz** | 1 | NOAEL/100 |
|  | 100 | NOAEL |
|  | 1000 | NOAELx10 |
| **Mixture I** |  |  |
| **Cyproconazole + Epoxiconazole** | 1 | NOAEL/100 |
|  | 0.9 |  |
| **Cyproconazole + Epoxiconazole** | 100 | NOAEL |
|  | 90 |  |
| **Cyproconazole + Epoxiconazole** | 1000 | NOAELx10 |
|  | 900 |  |
| **Mixture II** |  |  |
| **Cyproconazole + Epoxiconazole + Prochloraz** | 1 | NOAEL/100 |
|  | 0.9 |  |
|  | 1 |  |
| **Cyproconazole + Epoxiconazole + Prochloraz** | 100 | NOAEL |
|  | 90 |  |
|  | 100 |  |
| **Cyproconazole + Epoxiconazole + Prochloraz** | 1000 | NOAELx10 |
|  | 900 |  |
|  | 1000 |  |
