# Supplementary material for: Hepatotoxic combination effects of three azole fungicides in a broad dose range
Source: Arch Toxicol. 2017 Oct 16;92(2):859–72. doi: 10.1007/s00204-017-2087-6 (PMC5818588; doi:10.1007/s00204-017-2087-6)
Supplement: Supplementary file 2 — Supplementary material 2 (DOC 33 kb) Supplementary Table 2: Primers used to specifically amplify particular rat genes [file 204_2017_2087_MOESM2_ESM.doc]

**Table 2** Primers used to specifically amplify particular rat genes via quantitative real time PCR

| Gene | Forward Primer (5´- 3`) | Reverse Primer (5´- 3`) |
| --- | --- | --- |
| *Cyp1a1* | TTCACCATCCCCCACAGCACCATA | CAGGCCGGAACTCGTTTGGATCAC |
| *Cyp2b1* | ATGGAGAAGGAGAAGTCGAACC | CTTGAGCATCAGCAGGAAACC |
| *Cyp3a1* | CCCAGCTAGAGGGACAACAC | CGGGTCCCAAATCCGTAGAG |
| *GAPDH* | CCGTGGGGCAGCCCAGAAC | GCCCCAGCATCAAAGGTGGAGGA |
| *β-Actin* | AGGGAAATCGTGCGTGAC | CGCTCATTGCCGATAGTG |
