# Supplementary material for: Hepatotoxic combination effects of three azole fungicides in a broad dose range
Source: Arch Toxicol. 2017 Oct 16;92(2):859–72. doi: 10.1007/s00204-017-2087-6 (PMC5818588; doi:10.1007/s00204-017-2087-6)
Supplement: Supplementary file 3 — Supplementary material 3 (DOCX 45 kb) Supplementary Table 3: Effects on gene expression by cyproconazole, epoxiconazole and prochloraz and the two substance combinations as obtained by molecular toxicity pathway finder RT2 profiler PCR array for the dose level NOALx10. Of the 384 genes analysed on the array only those are listed here for which fold induction or repression by more than factor 2 with a significance of p < 0.05 was found in any of the treatment groups, and these expression changes are printed bold [file 204_2017_2087_MOESM3_ESM.docx]

| **Gene**  Supplementary table 3 | **Cyproconazole**  **(mean 2^-ΔΔCt^)** | **Epoxiconazole**  **(mean 2^-ΔΔCt^)** | **Prochloraz**  **(mean 2^-ΔΔCt^)** | **Mixture I**  **(mean 2^-ΔΔCt^)** | **Mixture II**  **(mean 2^-ΔΔCt^)** |
| --- | --- | --- | --- | --- | --- |
|  | **1000 ppm** | **900 ppm** | **1000 ppm** | **1000:900 ppm** | **1000:900:1000 ppm** |
| Abcb11 | 1.79** | 1.65 | 1.78** | **2.40**** | **2.10**** |
| Abcb1a | **7.79**** | **5.75*** | **3.45*** | **12.60**** | **9.99**** |
| Abcb1b | **5.89*** | **19.42** | **2.46** | **2.21** | **4.11*** |
| Abcc1 | 1.66* | 1.78 | 1.28 | **2.52*** | 1.83* |
| Abcc2 | **2.69*** | **3.77*** | **2.52**** | **5.39*** | **3.96**** |
| Abcc3 | **74.16**** | **71.82*** | **14.57*** | **94.70**** | **100.47**** |
| Abl1 | 1.71 | 1.87 | 1.49 | **2.49**** | **2.72*** |
| Acaa1a | 1.95 | **2.46*** | **2.03** | 1.92 | 1.69 |
| Acaca | 1.58** | **3.44**** | 1.08 | **2.77*** | 1.93** |
| Acad9 | 1.15 | **2.26**** | 1.20 | 1.74 | **2.23*** |
| Acat3 | 1.58* | 1.59* | 1.65** | **2.52**** | **2.22*** |
| Acly | **2.13**** | **4.54**** | 1.30 | 2.00** | **2.12*** |
| Aco1 | 1.79* | 1.79* | 1.66* | **2.66**** | **2.29**** |
| Aco2 | 1.34 | 1.94* | 1.47 | **2.13*** | 1.92* |
| Acot2 | 1.27 | 1.36 | 1.29 | 1.43 | **2.04*** |
| Acot3 | 1.31 | 0.81 | 1.23 | 1.61 | **2.53**** |
| Acot8 | 1.35 | 1.69 | 1.59* | **2.49*** | **2.20** |
| Acot9 | **2.31*** | 1.88* | 1.32* | **2.37*** | **2.99**** |
| Adh1 | 0.74 | **0.47**** | 0.85 | 1.03 | 0.90 |
| Ahr | **3.07**** | **3.60**** | **2.44** | **3.52*** | **2.97*** |
| Aldh1a1 | **22.68**** | **13.02**** | **5.04*** | **22.99**** | **26.18**** |
| Apex1 | **2.37**** | 1.81* | **2.89**** | **3.12**** | **3.20**** |
| Apof | 0.86 | 1.59 | **3.01*** | **3.70**** | 1.97 |
| Asns | **2.07** | 1.21 | 0.95 | **9.09*** | **9.26**** |
| Atf6 | 1.60* | 1.84* | 1.88* | **2.54**** | 1.90* |
| Atm | **2.33*** | **2.53**** | **2.66**** | **2.90**** | **2.78*** |
| Atp8b1 | **3.42**** | **3.98**** | **3.52**** | **4.98**** | **3.32** |
| Bcl2l1 | 1.63 | **2.20*** | **2.17**** | **2.51*** | 1.96* |
| Bcl2l11 | 1.53 | **3.09** | **2.38**** | **3.93*** | **2.37** |
| Birc3 | 1.44 | 1.53 | 0.94 | **2.49*** | 1.77* |
| C9 | 1.84* | **2.02*** | 1.25 | 1.70* | 1.26 |
| Casp2 | 1.39 | **2.11*** | **2.14** | **2.24** | 1.78 |
| Casp7 | 1.83** | **2.42*** | **2.15*** | **2.98**** | **2.62*** |
| Cd36 | **2.95*** | 1.46 | 1.92* | **2.41*** | **3.08**** |
| Cd8a | 1.19 | 0.59 | 0.97 | **2.93*** | 1.07 |
| Cdkn1a | 0.95 | 0.72 | **0.23**** | **0.07**** | **0.11**** |
| Ces2c | **6.62**** | **5.63**** | **2.65** | **14.95**** | **14.40**** |
| Comt | 1.39 | 1.94 | 1.11 | **2.33**** | **2.15*** |
| Cpt1a | 1.18 | 1.70 | 0.96 | **2.93*** | 1.76 |
| Cpt2 | 1.42 | 1.29 | 1.91* | **2.67*** | **2.84**** |
| Ctsb | 1.76* | **2.15*** | 1.33 | 1.79 | 1.43 |
| Ctse | 1.72 | 1.80 | **2.61*** | **2.46**** | **2.17** |
| Cyp1a1 | **26.64** | **25.03** | **405.03**** | **398.85**** | **690.41**** |
| Cyp1a2 | 0.88 | 1.39 | **3.26** | 1.90 | **2.10*** |
| Cyp2b2 | **87.40**** | **76.21**** | **17.03**** | **140.05**** | **172.46**** |
| Cyp2c11 | 1.35* | **2.32**** | 1.22 | 1.73* | 1.71 |
| Cyp2c37 | **4.20**** | **5.83**** | **2.40**** | **6.41*** | **5.20**** |
| Cyp2d4 | **2.45** | **3.52*** | **0.21** | 1.09 | **2.85** |
| Cyp2e1 | 1.21 | 1.48 | 1.85 | **2.08**** | 1.56 |
| Cyp3a2 | **4.52*** | **5.06**** | **2.84*** | **5.47**** | **4.63**** |
| Cyp3a23/3a1 | **13.52**** | **6.52**** | **2.57**** | **26.13**** | **24.10**** |
| Cyp7a1 | 1.49 | **4.70** | **5.44*** | **8.89**** | **2.81** |
| Dhcr24 | **2.13*** | **2.12*** | 1.78 | **2.00*** | 1.59 |
| Diablo | **2.05**** | **2.46**** | 1.86* | **2.40**** | **3.05*** |
| Dlat | 1.79* | **2.33**** | 1.43* | 1.94* | 1.79 |
| Dnajb1 | **2.84** | **2.05**** | 1.82* | 1.83* | **2.55*** |
| Dnajc5 | 1.67 | 1.90* | **2.03*** | **2.40*** | 1.76* |
| Duox1 | **5.35** | **11.79** | **2.74** | **13.64** | **51.68*** |
| Duox2 | **2.52** | **5.09** | **2.07*** | **6.31*** | **12.58*** |
| Ehhadh | 1.61 | 1.88* | 1.07 | **2.30*** | **2.18**** |
| Eif2ak3 | 1.92* | **2.51**** | 1.85** | **2.56*** | **2.48**** |
| Ep300 | **2.41*** | **2.13*** | 1.85* | **2.44*** | **2.08**** |
| Ephx1 | **3.66**** | **4.17*** | **2.09** | **4.05**** | **5.42*** |
| Ercc1 | 1.74* | 1.98 | **3.23** | **2.13** | **2.19**** |
| Ercc2 | **2.74** | **2.27** | **2.67**** | **2.79** | 1.93* |
| Fas | **2.55**** | 1.89 | 1.62* | **2.19*** | **2.08**** |
| Fasn | **2.74**** | **4.99**** | 1.29 | 1.85 | 1.97 |
| Fmo2 | **0.38*** | 1.20 | 1.49 | **2.25*** | 1.75* |
| Fmo5 | 0.80 | 1.22 | 1.11 | **2.46*** | **2.51** |
| Fxc1 | 1.60* | 1.57* | 1.73** | **2.16** | **2.73**** |
| Gpd1 | 1.98** | **2.36**** | 1.46* | 1.92* | 1.72** |
| Gpx2 | **11.98*** | **2.05** | **2.14** | **8.47**** | **37.94** |
| Gsta5 | **6.60**** | **4.59** | **3.36*** | **8.74**** | **12.61**** |
| Hadha | 1.45* | 1.44* | 1.66** | **2.09**** | 1.77** |
| Herpud1 | 1.33 | **2.28*** | **2.42**** | **2.68**** | **2.66*** |
| Hpn | 1.96 | **2.24*** | 1.69 | 1.88 | 1.33 |
| Hsp90aa1 | 1.77 | 1.97* | 1.95 | 1.57 | **2.46*** |
| Hspb8 | **2.42*** | **2.88**** | **2.43**** | **3.09**** | **3.06**** |
| Hsph1 | 1.57 | 2.00** | **2.50** | 1.93* | **2.70*** |
| Icam1 | **2.05*** | 1.57 | 1.14 | 1.66 | 1.59 |
| Il1a | 1.75 | 1.24 | 1.53 | **2.99*** | 1.49 |
| Il1b | **0.45*** | 0.87 | 1.25 | 0.77 | 0.71 |
| Itgax | **3.21** | **2.55** | **2.65** | **6.46**** | **3.32** |
| Klf1 | 1.02 | 1.27 | **2.10*** | 0.90 | 0.94 |
| Lss | 1.66 | 1.78* | 1.43 | **2.70**** | 1.68* |
| Ly6d | 1.12 | 1.82* | **2.57**** | **12.20** | **8.56*** |
| Lyz2 | **2.49*** | 0.98 | 1.56 | **3.98*** | **2.22** |
| Manba | **2.79**** | 1.62* | 1.30* | **2.69**** | **2.53**** |
| Mdm2 | 1.74** | **2.01**** | 1.37* | **2.69**** | **2.71**** |
| Mlh1 | 1.46 | 1.34 | **2.09**** | 1.84 | 1.41 |
| Mlx | 1.30 | 1.60* | 1.69* | 1.92** | **2.04**** |
| Mrps18b | 1.64** | 1.67** | 1.74** | 1.72* | **2.12**** |
| Nfkb1 | 1.59* | 1.91** | 1.49* | **2.08*** | 1.56* |
| Nploc4 | **2.10**** | **2.58**** | **2.00**** | **2.65**** | **2.53**** |
| Nqo1 | **2.44*** | **3.06*** | 1.72 | **3.43**** | **3.82**** |
| Nr0b2 | 1.25 | **3.80** | **4.83** | **5.07*** | **3.28** |
| Nup210 | **2.02*** | 1.98 | 1.11 | **2.64** | **2.03** |
| Parp1 | **2.20*** | **2.23*** | 1.30 | 1.60 | 1.79 |
| Pdyn | **2.32** | **2.04** | **3.92**** | **5.42** | **5.82** |
| Por | **4.01**** | **4.74**** | **4.17*** | **11.62**** | **8.75**** |
| Ppara | 1.66 | 1.89* | 1.69 | **3.82*** | **2.80*** |
| Ppargc1a | **3.68**** | **2.78**** | **3.48**** | **7.14**** | **5.33**** |
| Pvr | **2.13**** | 1.65 | 1.38 | **2.17** | **2.79** |
| Sc4mol | **2.00*** | 1.65 | **2.63**** | **3.68**** | **3.48**** |
| Scd1 | 0.58 | 1.90 | **0.18*** | 0.64 | **0.45** |
| Slco1a4 | **3.96**** | **2.94**** | 1.78 | **4.86**** | **3.74**** |
| Spata2 | **2.02** | **2.36** | **2.28** | **2.82*** | **2.48*** |
| Stbd1 | **2.62**** | **2.75**** | **2.61**** | **3.69*** | **2.37** |
| Tff3 | **2.32*** | 1.60 | **2.94**** | **3.40** | 1.86 |
| Tnfrsf1a | 1.74* | **2.19*** | **2.60**** | 1.71 | 1.57 |
| Ubqln2 | **2.19**** | **2.15** | 1.72** | **3.18**** | **2.60**** |
| Ucp2 | 1.13 | **2.16*** | **2.24*** | 1.48 | 1.01 |
| Ugt1a1 | **4.12**** | **2.99**** | 1.70** | **8.09**** | **5.91**** |
| Ugt2b1 | **6.60**** | **6.06**** | **4.17**** | **11.16**** | **11.93*** |
| Xrcc1 | **2.18**** | 1.80* | 1.60* | **2.58**** | 1.77** |
| Xrcc5 | 1.79** | 1.61 | 1.31 | 1.51 | **2.42**** |

bold: foldchange > 2, * p < 0.05, ** p < 0.01
